# Role of Exercise as a Modulating Factor in Arrhythmogenic Cardiomyopathy

**DOI:** 10.1007/s11886-021-01489-0

**Published:** 2021-05-07

**Authors:** Alessandro Zorzi, Alberto Cipriani, Riccardo Bariani, Kalliopi Pilichou, Domenico Corrado, Barbara Bauce

**Affiliations:** Department of Cardiac, Thoracic, and Vascular Sciences and Public Health, University of Padova, Via Giustiniani, 2, 35128 Padova, Italy

**Keywords:** Athlete’s heart, Exercise prescription, Sports cardiology, Sudden death, Ventricular arrhythmias

## Abstract

**Purpose of Review:**

The review addresses the role of exercise in triggering ventricular arrhythmias and promoting disease progression in arrhythmogenic cardiomyopathy (AC) patients and gene-mutation carriers, the differential diagnosis between AC and athlete’s heart and current recommendations on exercise activity in AC.

**Recent Findings:**

AC is an inherited heart muscle disease caused by genetically defective cell-to-cell adhesion structures (mainly desmosomes). The pathophysiological hallmark of the disease is progressive myocyte loss and replacement by fibro-fatty tissue, which creates the substrates for ventricular arrhythmias. Animal and human studies demonstrated that intense exercise, but not moderate physical activity, may increase disease penetrance, worsen the phenotype, and favor life-threatening ventricular arrhythmias. It has been proposed that in some individuals prolonged endurance sports activity may in itself cause AC (so-called exercise-induced AC).

**Summary:**

The studies agree that intense physical activity should be avoided in patients with AC and healthy gene-mutation carriers. However, low-to-moderate intensity exercise does not appear detrimental and these patients should not be entirely deprived from the many health benefits of physical activity.

## Introduction

Arrhythmogenic cardiomyopathy (ACM) is an inherited heart muscle disease caused by genetically defective cell-to-cell adhesion structures (mainly desmosomes) [1]. The pathophysiological mechanism of ACM is a progressive myocyte loss and substitution by fibro-fatty scar, which represents the substrate for re-entrant ventricular tachycardia (VT) and myocardial dysfunction [2]. Although wall motion abnormalities resulting from myocardial scarring are usually more evident in the thinner right ventricle (RV), cardiac magnetic resonance (CMR) studies demonstrated that ACM is often biventricular [3, 4•]. However, only in a minority of patients the degree of myocyte loss is severe enough to cause symptomatic ventricular dysfunction. Life-threatening ventricular arrhythmias (VAs), usually ventricular fibrillation (VF), and heart failure may also occur abruptly, during the so-called hot-phases, characterized by acute bouts of myocyte necrosis and inflammation similarly to acute myocarditis [5].

Sports activity is a well-known arrhythmic trigger and ACM is a leading cause of sudden death (SD) in the athlete [6–10]. More recently, intense exercise has been recognized as a modulating factor promoting phenotypic penetrance and disease progression both in animal studies and in familial ACM [11–15, 16••, 17–23]. Observation that some long-term endurance athletes may develop disease features or even overt ACM in the absence of gene mutations has led to the hypothesis that ACM may be entirely “exercise-induced” [24]. On the other hand, there is no demonstration that even low-to-moderate intensity exercise may be detrimental in patients with ACM or in their gene-positive family members and recent recommendations suggest not to deprive entirely these subjects from the many benefits of physical activity [25].

In the present article, we will review the role of exercise in triggering ventricular arrhythmias and promoting disease progression. We will also briefly address the differential diagnosis between ACM and athlete’s heart. Finally, we will discuss current recommendations about competitive sports and leisure time exercise in ACM patients.

## Animal Studies

The role of physical exercise as a trigger of arrhythmic events or disease progression factor has been evaluated in different animal models.

In 2006, Kirchhof and coworkers investigated the effects of age and endurance training on heterozygous plakoglobin-deficient (*JUP*+/−) mice [11]. They found that RV dilatation and dysfunction as well as VAs were exacerbated by daily swimming, supporting that endurance training could accelerate disease progression. However, neither cardiomyocyte abnormalities or fibrofatty replacement nor ultrastructural changes of desmosomes or adherens junctions were observed in trained mice. Further studies from the same group demonstrated that a load-reducing therapy (furosemide and nitrates) prevents training-induced disease development and progression as well as VAs in *JUP*-deficient (+/−) mice; however, these results have yet to be confirmed in the clinical setting [12].

The effect of endurance training was also assessed in a mouse model overexpressing a nonsense plakophilin-2 (*PKP2*) gene mutation [13]. Endurance training was associated with abnormal localization and distribution of the Cx43 and exacerbated by 5-fold the risk of developing RV dysfunction. In another study, endurance training was evaluated related to the gradient overexpression of the transgene carrying another nonsense *PKP2* gene-mutation [14]. In this case, exercise was associated with RV outflow tract dilatation but no electrocardiographic changes or higher inducibility of VAs. Noteworthy, also transgenic *PKP2* animals, similarly to the *JUP*-deficient mice, showed no histological/ultrastructural changes typical of ACM regardless training. Both these models suggest that exercise may induce ventricular dilation/dysfunction and favor VAs in mice with abnormal desmosomal proteins but do not cause the structural and ultrastructural changes typical of ACM, suggesting that the deleterious effects of exercise are potentially reversible.

In contrast, a recent study on a conditional heterozygous desmoplakin (*DSP*) mouse model showed that treadmill exercise partially rescued ACM phenotype by normalizing two-thirds of the aberrant regulated genes in sedentary haploinsufficient *DSP* mice, particularly those related to inflammation, epithelial-mesenchymal transition, and oxidative phosphorylation [15]. According to this model, treadmill exercise did not worsen cardiac systolic function or electrophysiologic parameters but was associated with eccentric cardiac hypertrophy and significant reduction of the apoptotic index in the presence of normally distributed intercalated discs proteins.

## ACM as a Cause of Exercise-Related Sudden Death

The recognition of ACM as a cause of exercise-related SD dates back to 1988. Thiene et al. found that 12 of 60 (20%) young (<35 year-old) victims of SD in the Veneto region of Italy had morphologic features of RV cardiomyopathy. Ten of the subjects had died during exertion [6]. Further studies on larger populations confirmed that ACM is one of the leading causes of SD in athletes of the Veneto region of Italy (14% of cases) and that athletes with ACM had 5.4 times higher risk of dying suddenly than their sedentary counterpart [7]. In a British study on SD in athletes, whose hearts were referred for post-mortem to a tertiary center, the prevalence of ACM was similar (13%) to that found in the Italian series [9]. Conversely, ACM accounted for only 6% of SD cases in the USA [26]. Such discrepancy may depend on one hand on the experience of the pathologist or coroner performing post-mortem analysis, who may miss this rare and peculiar disease, and on the fact that the US series included many black athletes who are rarely affected by ACM.

**Fig. 1:**
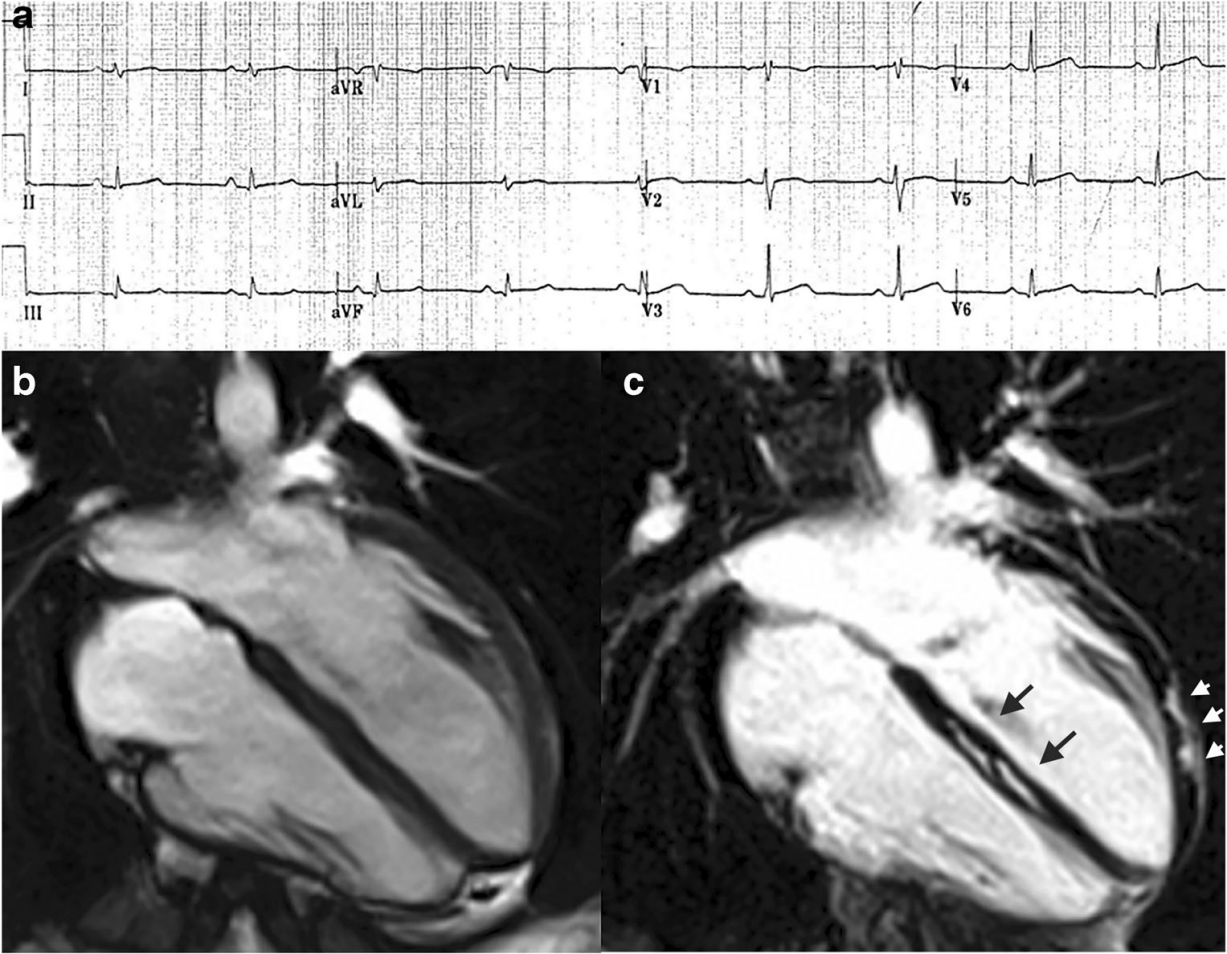
Representative case of left-dominant arrhythmogenic cardiomyopathy variant in a patient with a desmoplakin-gene mutation. The left-dominant arrhythmogenic cardiomyopathy variant may show an unremarkable electrocardiogram except for low-QRS voltages in the limb leads (**a**) and normal dimension and systolic function of both ventricles (end-diastolic frame of cine cardiac magnetic resonance sequence in long-axis 4-chamber view). **b** Post-contrast cardiac magnetic resonance images disclose myocardial fibrosis in the form of a late gadolinium enhancement stria in the epicardium of the left ventricular lateral wall (arrowheads) and midmural layer of the interventricular septum (arrows) (**c**) (reproduced with permission from: De Lazzari M et al. J Am Heart Assoc. 2018 Nov 20;7(22):e009855. doi: 10.1161/JAHA.118.009855) [27]

Systematic preparticipation screening has demonstrated to reduce the risk of SD from classic ACM variants (right-dominant or biventricular) because of its ability to identify early asymptomatic patients by investigating abnormal electrocardiogram (ECG) findings or VAs at exercise testing [8]. However, a subset of patients exhibits an ACM variant that predominantly affects the left ventricle (left-dominant). ECG abnormalities such as T-wave inversion in the lateral leads V4-V6 and low QRS voltages in the limb leads and left ventricular regional systolic function abnormalities may be observed, but standard clinical investigations are often normal and the diagnosis requires demonstration of subepicardial/midmyocardial late enhancement on cardiac magnetic resonance (Fig. 1) [28]. For this reason, the incidence of SD from classic ACM has markedly decreased since the introduction of pre-participation screening, while the difficulty to diagnose “left dominant” variant had led to the fact that this clinical entity is now an increasingly reported substrate of SD [29]. According to recent studies, exercise testing for evaluation of VAs may provide additional value for raising the suspicion of an underlying left-dominant ACM [30]. In particular, the occurrence of exercise-induced repetitive premature ventricular beats with a right-bundle-branch block and superior axis pattern (suggesting an origin from the infero-lateral left ventricular wall) may deserve more in-depth investigation by cardiac magnetic resonance [31].

## Role of Sports Activity in Promoting Disease Penetrance and Progression

**Fig. 2:**
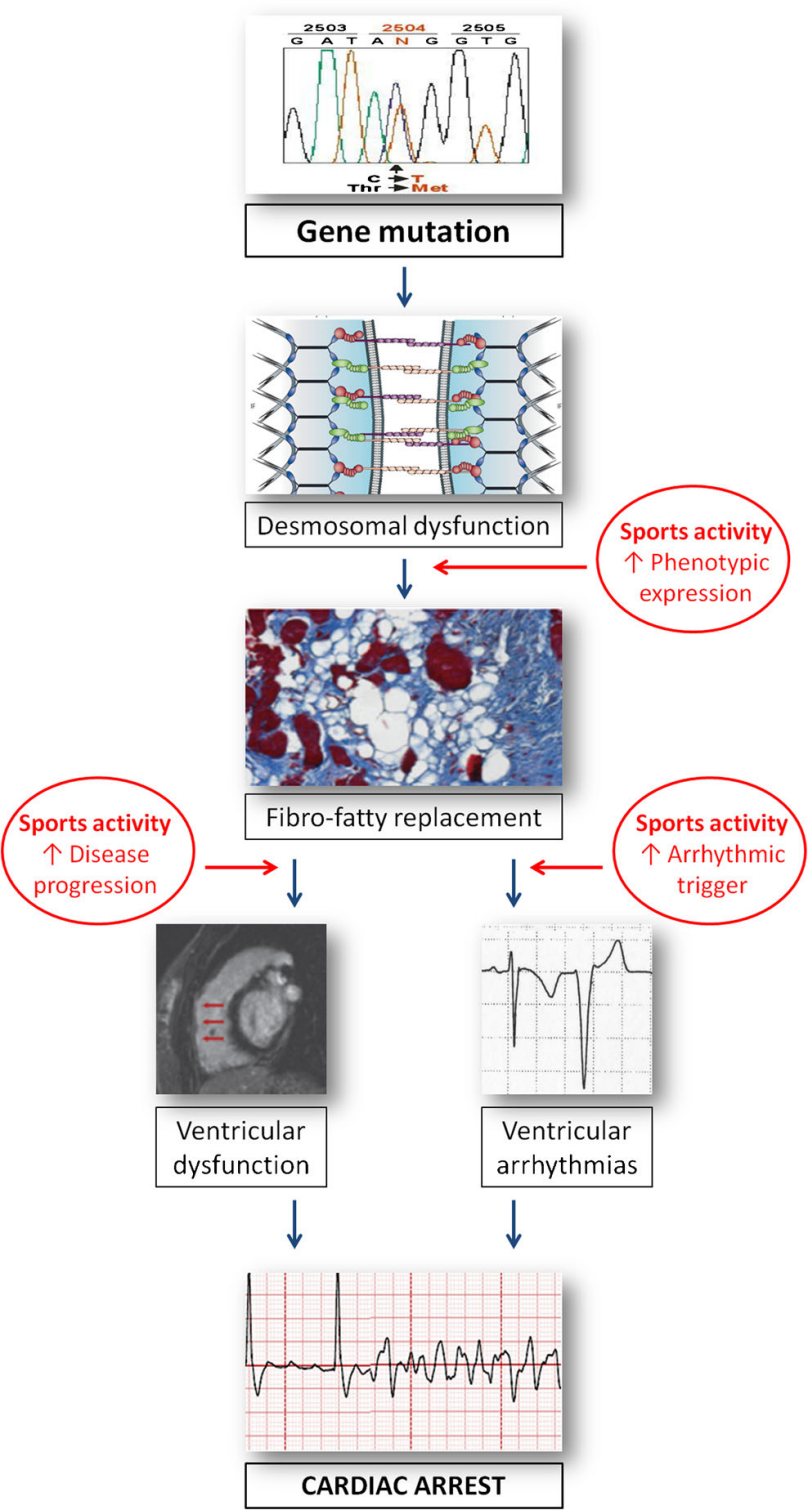
Role of intense sports activity in the natural history of arrhythmogenic cardiomyopathy, from desmosomal-gene mutation to phenotypic expression and cardiac arrest due to ventricular fibrillation. Sports activity may promote development of phenotypic expression, accelerate disease progression, and trigger life-threatening ventricular arrhythmias (reproduced with permission from: Corrado D, Zorzi A. Eur Heart J 2015 Jul 14;36(27):1708-10. doi: 10.1093/eurheartj/ehv183. Epub 2015 May 12, with permission from Oxford University Press) [32]

**Fig. 3:**
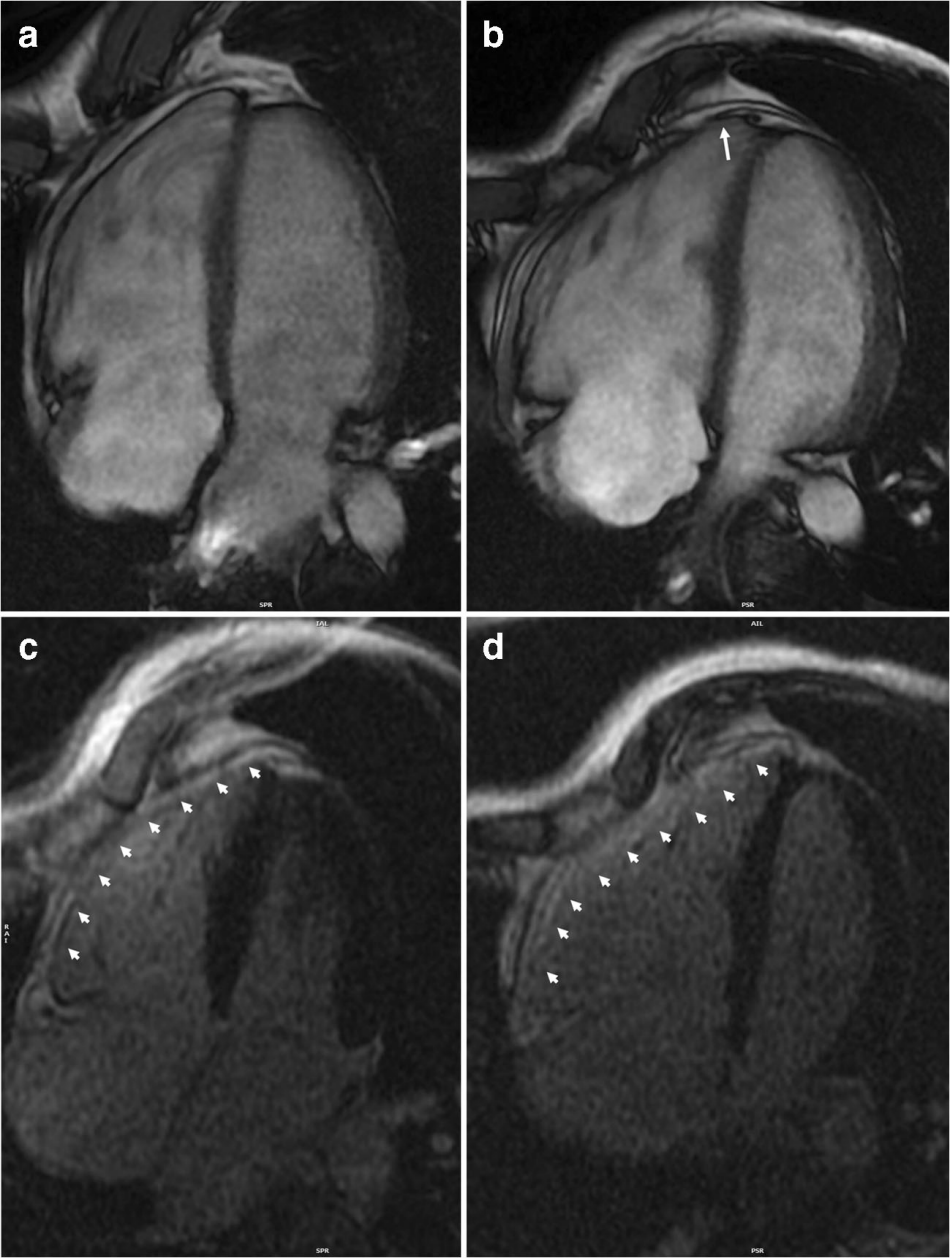
Representative example of the role of endurance exercise in worsening the arrhythmogenic cardiomyopathy phenotype. A 38-year-old runner received a diagnosis of arrhythmogenic cardiomyopathy after investigation of ventricular arrhythmias and ECG abnormalities at preparticipation screening. Genetic testing revealed the presence of a plakophilin-2 gene mutation. At the time of diagnosis, cardiac magnetic resonance 4-chamber view on cine sequences found dilatation of the right ventricle with mild dysfunction (**a**) and diffuse right ventricular late-enhancement on post-contrast sequences (arrowheads) (**b**). Although according to the Italian law he was considered not eligible for competitive sports activity, he continued to practice high-intensity endurance training. After 4 years, a new cardiac magnetic resonance showed a more enlarged right ventricle, an apical aneurysm (arrow), and a moderate right ventricular dysfunction (**c**). Post-contrast sequences confirmed the presence of a diffuse right ventricular late enhancement (arrowheads) (**d**)

Table 1 summarizes the design and the results of the studies that addressed the role of exercise activity in modifying the clinical course of patients with ACM and in genotype positive/phenotype negative individuals [16••, 17–23] The studies agree that intense and prolonged physical activity: (1) favors the development of the disease in genotype positive/phenotype negative patients; (2) worsen the degree of ventricular dysfunction in patients with overt ACM; (3) increases the likelihood of sustained ventricular arrhythmias and implantable cardioverter-defibrillator (ICD) interventions (Figs. 2 and 3). Common limitations of these studies included their retrospective nature, the fact that the type and amount of exercise was self-reported and that the definition of type and amount of exercise was heterogeneous.

**Table 1:** Main human studies addressing the role of sports activity as a modifying factor in arrhythmogenic cardiomyopathy

Reference	Aim and design of the study	Study population and definitions	Main results	Conclusions
James CA et al. J Am Coll Cardiol. 2013 [16••]	Aim: to test the hypothesis that exercise influences age-related penetrance, arrhythmic risk, and progression to heart failure in ACM Study type: Observational, retrospective.	Study population: 87 patients with pathogenic ACM associated desmosomal mutation (76 PKP2, 7 DSG2, 3 DSP, 1 DSC2) Definitions: “Endurance athletics” were sports with a high dynamic demand (>70% Max O_2_), done for at least 50 h/year at vigorous intensity.	The 56 ACM endurance athletes: 1. Developed symptoms at a younger age 2. Had a lower lifetime event free survival from VT/VF 3. Had a lower lifetime event free survival from HF	The amount and intensity of exercise increases the likelihood of diagnosis, of VT/VF, and of developing HF among ACM desmosomal mutation carriers
Sawant ACM et al., J Am Heart Assoc. 2014 [17]	Aim: To investigate whether exercise is associated with onset of gene-elusive ACM and has a differential impact in desmosomal and gene-elusive patients. Study type: Observational, retrospective.	Study populations: ACM patients who met revised 2010 ACM Task Force Criteria. Definitions: “Endurance athletics” were sports with a high dynamic demand (>70% Max O_2_), done for at least 50 h/year at vigorous intensity.	1. ACM patients without desmosomal mutations did significantly more intense exercise prior to presentation than desmosomal mutation carriers 2. Patients without desmosomal mutations were significantly less likely to have a family history of ACM 3. Exercise history influences disease course more in gene-elusive ACM patients. Top intensity exercise was associated with significantly earlier age of onset, worse structural disease at clinical presentation, and shorter freedom from ventricular arrhythmia in follow-up	Gene-elusive, non-familial ACM is associated with very high intensity exercise, suggesting exercise has a disproportionate role in the pathogenesis of these cases
Saberniak J et al. Eur J Heart Fail 2014 [18]	Aim: To investigate the impact of vigorous exercise on myocardial function in patients with ACM and in their mutation positive family members. Study type: Observational, cross-sectional.	Study populations: 110 patients, 65 ACM index patients and 45 mutation-positive family members. Definitions: Intensity of physical activity was graded as vigorous if ≥6 METs. The duration of regularly performed exercise was expressed in years. Subjects with a history of physical activity with intensity ≥6 METs for ≥4 h/week (≥1440 METs × min/week) for ≥6 years were defined as athletes.	ACM patients with a history of athletic activity, compared with ACM non-athletes, had: 1. Reduced RV and LV function 2. Worse outcome, with a higher frequency and earlier onset of VA 3. Need of cardiac transplantation	ACM subjects with a history of athletic activity showed reduced RV and LV function compared with non-athletes by echocardiography and by MRI. Higher levels of physical activity correlated with reduced LV and RV function, and only athletes had cardiac transplantation. Diagnosis of ACM and onset of VA occurred at younger age in athletes compared with non-athletes
Ruwald ACM et al., Eur Heart J 2015 [19]	Aim: To assess the effects of competitive and recreational sport participation on age at first onset of symptoms, and risk of VT/death in ACM probands. To investigate whether a change in exercise level after ACM diagnosis is associated with a reduction in the risk of subsequent VT/death. Study type: Observational, prospective	Study population: 322 ACM probands or family members Definitions: Patients were asked what type of exercise level they performed both before and after diagnosis, with the following pre-specified fixed options: (i) inactive; (ii) recreational; (iii) competitive /professional; or (iv) unknown. Based on the recommendations for sports participation in patients with cardiovascular disease they differentiate between high dynamic sports (basketball, soccer, hockey, skiing, running, biking, and tennis) and low to moderately dynamic sports (bowling, golf, weight lifting, wrestling, baseball, or softball)	ACM patients engaged in competitive sport had: 1. Significantly higher risk of VT/death 2. Symptoms developed at an earlier age	In ACM patients, competitive sport was associated with a twofold increased risk of VT/death, and earlier presentation of symptoms, when compared with inactive patients, and to patients who participated in recreational sport
Lie OH et al. JACMC EP 2018 [20]	Aim: to explore the impact of exercise intensity, exercise duration, and exercise dose on outcome in ACM patients Study type: Observational, retrospective	Study population: 173 patients diagnosed with ACM Definitions: Patients were classified as regularly engaging in high intensity exercise (>6 METs), for example, running, aerobics, fast swimming, or competitive sports, or low-intensity exercise (3 to 6 METs), for example, walking, dancing, or weight lifting. Regular physical activity <3 METs was not recorded as exercise. On the basis of exercise intensity and duration, they categorized patients into 4 groups: 1. Low-intensity and short-duration exercise (low/short); 2. Low-intensity and long-duration exercise (low/long); 3. High-intensity and short-duration exercise (high/short); 4. High-intensity and long-duration exercise (high/long)	1. VA were more prevalent in patients with high-intensity exercise than low-intensity exercise, and more prevalent in long-duration than short-duration exercise 2. High-intensity exercise was a strong and independent marker of VA, even when adjusted for the interaction with long-duration exercise, whereas long-duration exercise was not	High-intensity exercise and long-duration exercise were both associated with unfavorable outcome in ACM patients, but high-intensity exercise was a strong marker of VA independently from exercise duration. Low intensity exercise, even for long durations, was associated with a milder phenotype and can be advised for patients with ACM
Salas AR et al. J Cardiovasc Electrophysiol. 2018 [21]	Aim: To discover the impact of dynamic physical activity on 36 patients with high-risk definite ACM. Study type: Observational retrospective cohort study	Study population: 36 high-risk ACM Definitions: According to the guidelines on sports participation for patients with cardiovascular disease the following were considered high dynamic sports: badminton, long or middle-distance running (marathon), cross-country skiing, squash, basketball, ice hockey, hockey, rugby, football, swimming, singles tennis, handball, boxing, kayaking, cycling, decathlon, rowing, speed skating, and triathlon. The intensity of dynamic activity was classified in accordance with the mean frequency of weekly physical exercise sessions in the 10 years before diagnosis: high/competitive (>3 h/wk), moderate (1 to 3 h/wk), and minimal/inactive (<1 h/wk)	The major arrhythmic event-free survival was shorter and the occurrence of severe RV dysfunction was more probable in the high-intensity exercise group, followed by the moderate intensity group and by the low-intensity/inactive group	A dose-dependent relationship between a history of dynamic physical activity and phenotypic severity based on the early onset of major arrhythmic events and deterioration of RV contractility was observed.
Müssingrbrodt Int J Sports Med 2019 [22]	Aim: To assess the role of exercise on long-term results of radiofrequency CA therapy of VT in patients with ACM Study type: Observational	Study population: 38 patients with definite ACM and previous CA for VT. Before CA, 30 were involved in sports activities (recreational and competitive) and 8 had sedentary lifestyle Definitions: According to the recommendations for competitive athletes with cardiovascular abnormalities, highly dynamic sports such as running or cycling were distinguished from low to moderately dynamic sports such as walking, golf or yoga	After CA: 1. No patient continued competitive sport 2. Patients practicing recreational exercise activities were not exposed to a greater risk for VT than patients with a sedentary lifestyle 3. Patients with a sedentary lifestyle or low to moderately dynamic sports activities had the same risk for VT recurrence as patients with highly dynamic sports activities	Recreational sports do not impair long-term results after CA therapy compared with a sedentary lifestyle and are not associated with an increased risk for VT recurrences after CA
Paulin heart rhythm 2020 [23]	Aim: To evaluate the impact of exercise on arrhythmic risk and cardiac death in TMEM43 p.S358L ACM. Study type: Observational	Study population: 80 individuals with the TMEM43 p.S358L mutation enrolled in a prospective registry who had received a primary prevention ICD. Definitions: The modified Paffenbarger Physical Activity Questionnaire was used. Subjects were asked questions pertaining to daily walking distances; number of flights of stairs climbed; frequency, duration, and type of sports played; and leisure and recreational activity. MET/h per day were calculated.	Exercise ≥9.0 MET-h/day (high level) in the year before ICD implantation was associated with an adjusted 9.1-fold increased hazard of first appropriate ICD discharge.	Exercise ≥9.0 MET-h/day is associated with an increased risk of malignant ventricular arrhythmias in the TMEM43 p.S358L subtype of ACM.

*ACM* arrhythmogenic right ventricular cardiomyopathy, *CA* catheter ablation, *HF* heart failure, *ICD* implantable cardioverter-defibrillator, *LV* left ventricle, *MET* metabolic equivalent of task, *MRI* magnetic resonance imaging, *RV* right ventricle, *VA* ventricular arrhythmias, *VF* ventricular fibrillation, *VT* ventricular tachycardia

Many young patients with ACM may wish to participate in recreational and leisure time exercise activity, given the recognized beneficial effects of a physically active lifestyle. On the other hand, a sedentary behavior may carry adverse consequences (e.g., obesity, depression, social deprivation, increased risk of coronary artery disease) that potentially outweigh the benefits. It is difficult to infer from the majority of the studies whether there is a dose-response correlation between exercise and adverse outcome (i.e., ideally ACM patients should not exercise at all) or if there is a threshold of exercise type, intensity and/or duration that can be considered safe. However, the investigations that specifically addressed the outcome of ACM patients engaged in low-to-moderate intensity exercise found that their outcome was similar to that of sedentary patients. These findings suggest that not all exercise is the same and that patients with ACM may be able to derive the benefits of leisure time exercise without excess risk.

## The Theory of Exercise-Induced ACM

So far, we have discussed the potential role of exercise in modifying the natural history of patients with ACM or of their genotype-positive/phenotype-negative relatives. It has been proposed that intense and prolonged endurance exercise may cause ACM in the absence of gene mutations, so-called exercise-induced ACM. This theory was first advanced by Heidbuchel et al. in 2003, who described a series of 46 endurance athletes (predominantly cyclists) presenting with VAs. Because of associated abnormalities on electrocardiogram, Holter monitoring, and cardiac imaging, 60% met the criteria for definite ACM and 30% for probable ACM but only 1 athlete had a family history suggestive of ACM. During follow-up, almost 40% experienced major arrhythmic events [33]. Subsequent genotype-phenotype studies showed that ACM features in subjects who engaged in high-intensity exercise were typically not associated with a negative molecular genetics analysis for desmosomal gene mutations (so-called gene-elusive patients) ACM [17, 34]. In the “John Hopkins registry,” gene-elusive patients, particularly those <25 year-old, reported performing significantly more intense exercise prior to their diagnosis compared to desmosomal-gene mutation carriers [17]. Another analysis of the same registry, which focused on the effect of physical activity on ICD carriers, found that exercise restriction reduced the arrhythmic risk more in gene-elusive patients than in those with desmosomal mutations [35]. These observations further support the hypothesis that ACM may be acquired through intense exercise. Others reported that athletes may exhibit arrhythmogenic ventricular abnormalities typical of ACM variants such as isolated non-ischemic left ventricular scar or a particular form of RV outflow tract tachycardia associated with epicardial scar [36–38].

Taken together, these findings suggest that even in the absence of desmosomal-gene mutations, the increased hemodynamic load induce by prolonged intense exercise may be enough to cause permanent myocardial damage in some cases. However, it has to be noted that this phenomenon is rare as many studies demonstrated a lack of long-term adverse remodelling in large series of top-level athletes [24]. For this reason, it is plausible that a combination of a genetic predisposition that remains unknown and environmental factors (including exercise) is needed for an athlete with no mutations in known ACM-causing genes to develop the disease.

## Sports Activity in ACM Patients with an ICD

**Fig. 4:**
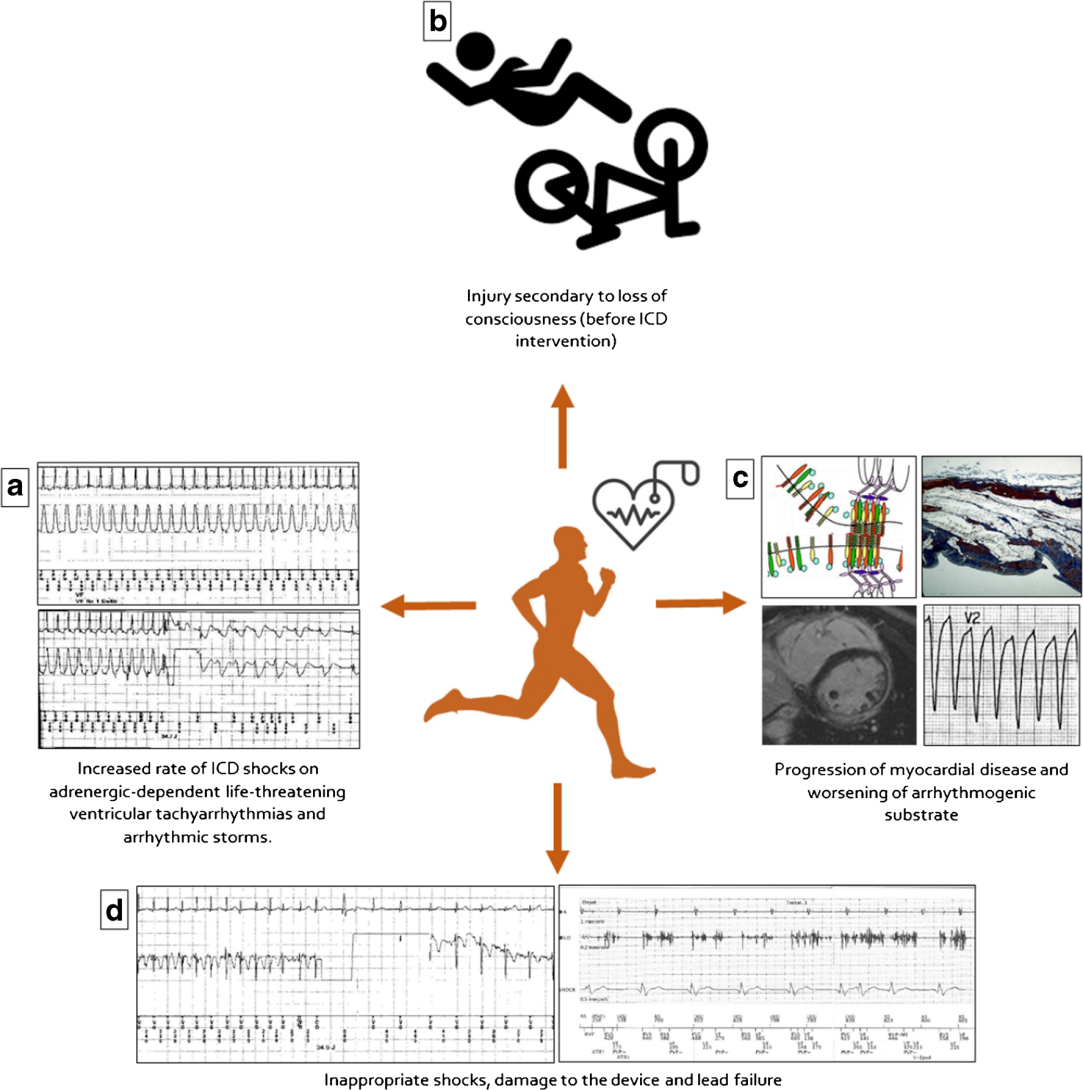
Potential unfavorable effects of sports activity in patients/athletes with an ICD. Sports in ICD carriers are potentially associated with an increased risk of: adrenergic-dependent ventricular tachyarrhythmias (**a**); athlete’s injury caused by loss of consciousness due to a syncopal tachyarrhythmia before the ICD intervention (**b**); development of phenotypic expression and accelerate disease progression of the underlying arrhythmogenic cardiomyopathy (**c**); inappropriate shocks caused by supraventricular tachycardia during sports activity (**d**, left) or by lead fracture with ventricular channel oversensing (**d**, right) (reproduced with permission from: Corrado D et al. Eur J Prev Cardiol. 2019 May;26(7):760-763. doi: 10.1177/2047487318805584. Epub 2018 Oct 18, with permission from Oxford University Press) [39]

The risk of SD during exercise is the main reason why patients with ACM are advised against engaging in sport activities. The ICD may offer protection against SD and may theoretically allow safe sport participation of athletes with ACM. Traditionally, there has been concern that intense exercise may influence the efficacy and safety of ICD therapy and increase the risk of inappropriate shocks, damage to the device, and lead failure [39] (Fig. 4). A multinational registry that recruited 372 athletes provided reassuring data about sports participation of ICD carriers by showing no arrhythmic deaths, resuscitated cardiac arrests, or shock-related injuries during follow-up and no increased risk of technical malfunction. On the other hand, the study confirmed the detrimental effect of competitive sports on the arrhythmic outcome of ACM patients by showing that an underlying ACM was the only variable associated with exercise-induced ICD shock [40•]. A sub-analysis on the arrhythmic outcome of ICD carriers enrolled in the European arm of the Registry who engaged in leisure time physical activity (mostly endurance disciplines such as running or cycling) showed that the risk of receiving appropriate ICD interventions was lower (6.3% vs. 20.2%) than their counterpart of competitive athletes. Also, in this subgroup, however, 2 of 3 patients that experienced appropriate ICD interventions during exercise were affected by ACM [41].

The association between continuing to practice high intensity exercise after ICD implantation and worse arrhythmic outcome was demonstrated by a study on 129 patients enrolled in the Johns Hopkins ACM registry, of whom 80% were athletes engaged in intense sports activity before the diagnosis (>936 METs*Hours yearly) [35]. More than half of patients (66/129) received appropriate ICD therapy during a median follow-up of 5.1 years. The chance of experiencing an appropriate intervention was directly related with the reduction of physical activity compared with baseline, and the correlation between exercise dose reduction and arrhythmic outcome was strengthen after correction for demographics, exercise dose before presentation, and established arrhythmic risk factors. The hazard ratio of 0.14 at multivariate analysis translated into an 86% lower risk of ICD therapy of those who reduced the exercise dose the most compared to those who reduced the least, independently from other variables. Of note, reducing the exercise “dose,” which takes into account the intensity of the activity, demonstrated higher effect in decreasing arrhythmic risk than just maintaining the same intensity for a shorter duration.

Finally, it should be emphasized that the reasons for competitive sports restriction in young patients with a prophylactic ICD go beyond the increased arrhythmic risk. As discussed, intense exercise plays a role in disease progression by accelerating the process of myocyte death and fibro-fatty replacement, thus worsening ventricular dysfunction.

In conclusion, considering the above-discussed evidence, there is general agreement that the implant of an ICD should not be considered a justification for participation of patients with ACM in competitive sports or high-intensity noncompetitive physical activities, particularly in high-risk patients. On the other hand, compliance or not to exercise restriction should be considered as an important factor in the decision to implant an ICD in intermediate-risk patients.

## Differentiating ACM and Athlete’s Heart

**Fig. 5:**
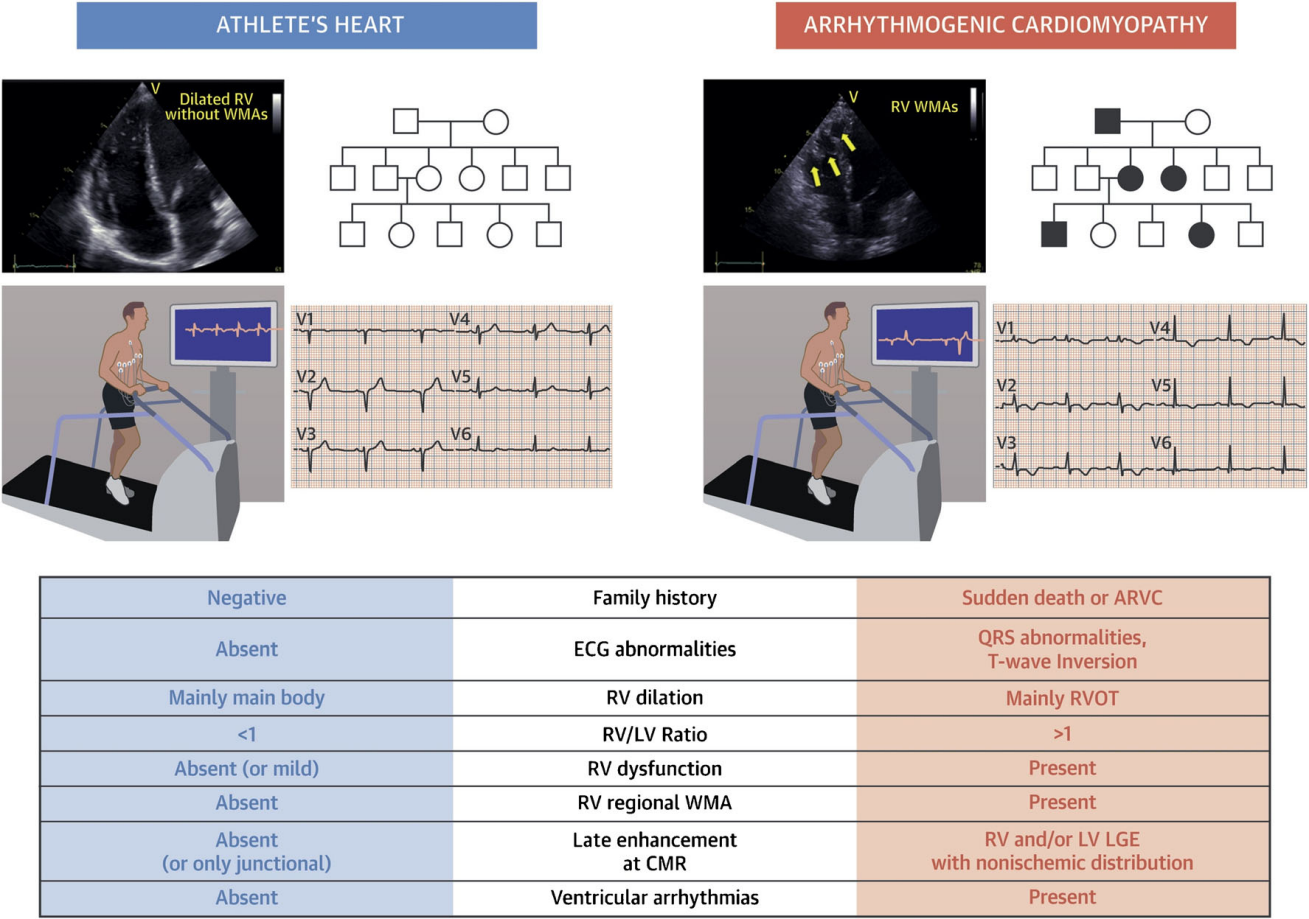
Differential diagnosis between arrhythmogenic cardiomyopathy and athlete’s heart. The differential diagnosis between athlete’s heart and arrhythmogenic right ventricular cardiomyopathy includes imaging features and comprehensive clinical approaches including ECG, ambulatory ECG monitoring, stress testing, and evaluation of family members. CMR, cardiac magnetic resonance; ECG, electrocardiography; LGE, late gadolinium enhancement; LV, left ventricular; RV, right ventricular; RVOT, right ventricular outflow tract; WMA, wall motion abnormality (reproduced with permission from: D’Ascenzi F et al. JACC Cardiovasc Imaging. 2018 Sep;11(9):1327-1339. doi: 10.1016/j.jcmg.2018.04.031, with permission from Elsevier) [53]

The long-running Italian experience with pre-participation screening demonstrated that early identification of affected individuals and restriction from competitive sports activity can substantially reduce the risk of SD [8, 42]. The electrical and structural remodelling of the athlete’s heart may sometimes require differential diagnosis with ACM. Although a detailed analysis of all elements useful for differentiating the two conditions goes beyond the scope of the review, some key points are:

1. ACM is an inherited disorder and family history of cardiomyopathies, and premature SD should be extended beyond first-degree family members. It is noteworthy that end-stage ACM can be confused with dilated cardiomyopathy [43].
2. Structural remodelling of the athlete’s heart may cause RV dilation (mainly the main body rather than the outflow tract) that parallels left ventricular dilation (right/left ventricular ratio remains <1) and is never accompanied by regional wall motion abnormalities. Mild global dysfunction may be observed at rest in highly trained athletes, but exercise echocardiography demonstrates normalization of contractility [44–46].
3. At cardiac magnetic resonance, only the presence of late-enhancement at the insertion points between the right and left free wall and the septum (junctional late enhancement) can be considered normal in athletes [37].
4. VAs, at rest or during exercise, are observed in a minority of athletes versus the majority of ACM patients [47–50]. Arrhythmias at exercise testing were also observed in the majority of asymptomatic desmosomal gene-mutation carriers without structural changes at cardiac magnetic resonance suggesting that VAs may be the first manifestation of ACM [51]. Besides its diagnostic value, exercise testing appears particularly useful for evaluating the threshold of VAs onset when prescribing moderate-intensity exercise in ACM patients.
5. Although multiple ECG abnormalities may be observed in ACM, T-wave inversion in the anterior leads V1-V4 is the most common and should always raise the suspicion of an underlying disease. In the athlete, anterior T-wave inversion should be considered normal only before pubertal development (“juvenile pattern of repolarization”) or in black individuals when preceded by J-point/ST-segment elevation (early repolarization variant typical of Afro-Caribbean athletes) [52].
6. The evaluation of an athlete should be multi-parametric: although no single test is diagnostic of ACM, the association of more than one abnormality at family history, resting, and exercise ECG and imaging increases the probability of a disease (Fig. 5).

## Competitive Sport, Leisure Time Physical Activity and ACM: Current Guidelines

Table 2 summarizes the recommendations for competitive sports participation and leisure time physical activity according to recent guidelines and consensus documents. They all agree that patients with ACM should not engage in competitive sport, with the possible exception of skills disciplines, or high-intensity leisure time physical exercise. According to the 2015 American Heart Association/American College of Cardiology guidelines, healthy gene carriers are not precluded from participation in competitive sports [54]. Conversely, more recent documents are more restrictive because of emerging evidence that regular exercise training and competitive sports can play a role in triggering cellular mechanisms leading to the development and progression of the disease phenotype in the presence of a predisposing gene abnormality [25, 55–57].

**Table 2:** Summary of recommendations for competitive sports participation and leisure time physical activity according to recent guidelines and consensus documents

Document	Recommendations for competitive sports	Recommendations for leisure time physical activity
2015 AHA/ACC competitive sports eligibility guidelines [52]	• No competitive sports (with the possible exception of skill sports) for possible, borderline or definite ACM • ICD placement for permitting participation in high-intensity sport not recommended	Not addressed
2015 International Task Force Consensus Statement on the Treatment of ARVC [54]	• No competitive sports in patients with definite ACM • Restriction from competitive sports activity may be considered in ACM family members with a negative phenotype, either healthy gene carriers or with unknown genotype	• Low-intensity exercise may be allowed
2019 EAPC recommendations for participation in competitive and leisure time sport in athletes with cardiomyopathies [55]	• No competitive sports in patients with definite or borderline ACM and gene positive/phenotype negative patients	• Low-intensity exercise recommended
2019 HRS guidelines on ACM [56]	• No competitive sports in ACM patients and gene positive/phenotype negative patients	• Frequent high-intensity endurance exercise not advised in ACM patients and gene positive/phenotype negative patients • Preference for low-intensity activities
2020 ESC guidelines sports cardiology and exercise in patients with cardiovascular disease [25]	• No competitive sports of any type in ACM patients and gene positive/phenotype negative patients	• Low-intensity exercise, 150 min/week, for all • Moderate intensity exercise only in low-risk patients* • High intensity not advised

*ACM* arrhythmogenic cardiomyopathy, *ACC* American college of cardiology, *AHA* American heart association, *EAPC* European association of preventive cardiology, *ESC* European society of cardiology, *ICD* implantable cardioverter defibrillator, *HRS* heart rhythm society

*Low risk = no history of cardiac arrest or ventricular tachycardia, low arrhythmic burden (<500 PVBs/day, no exercise-induced arrhythmias), minimal structural abnormalities

## Conclusions

There is compelling evidence from both animal and human studies that exercise is an important modulating factor in ACM that may favor disease penetrance in genotype positive/phenotype negative patients, worsen ventricular dysfunction, and trigger arrhythmias. For these reasons, there is unanimous consensus that ACM patients should be advised against participation in competitive sports (with the possible exception of skill disciplines at low-cardiovascular demands in selected cases) and high-intensity leisure time physical activity. On the other hand, ACM patients, particularly young with a mild disease, should not be deprived from the many health benefits of low-to-moderate intensity physical activity. In ACM, exercise is a medicine with a narrow therapeutic range and should be prescribed by experienced physicians on a case-by-case basis.
